# Non-uniform Excitation States in Photoinduced Deformation of Amorphous Carbon Nitride Films

**DOI:** 10.1038/s41598-018-33364-4

**Published:** 2018-10-10

**Authors:** Masami Aono, Tomo Harata, Nobuaki Kitazawa, Hiroshi Abe, Shingo Ishii, Yohei Sato, Masami Terauchi

**Affiliations:** 1 Department of Materials Science and Engineering, National Defense Academy, 1-10-20 Hashirimizu, Yokosuka, Kanagawa 239-8686 Japan; 2 Institute of Multidisciplinary Research for Advanced Materials, Tohoku University, 2-1-1 Katahira, Aoba-ku, Sendai, Miyagi 980-8577 Japan

## Abstract

Amorphous carbon nitride (a-CN_x_) films prepared via reactive radio frequency magnetron sputtering deform under on–off visible light illumination. We investigate the relationship between photoinduced deformation and surface electrical states via scanning electron microscopy with Ar^+^ laser irradiation (SEM-L). Two samples with different levels of photoinduced deformation are prepared. For the film with small photoinduced deformation, uniform secondary electron emission is observed on the film surface, regardless of whether the laser is on or off. On the a-CN_x_ film, which has fifty times larger photoinduced deformation than the previous film, light and dark patches, similar to a speckle pattern, appear on the film surface in SEM-L images. This anomalous phenomenon indicates non-uniformity of the electrical states excited by laser light irradiation. A size of the patches is well correlated with an inhomogeneous distribution of sp^3^C and sp^2^C, *I*_sp3C_/*I*_sp2C,_ obtained using soft X-ray emission spectroscopy (SXES). Simultaneously, temporal decrease in the sp^3^C component under illumination is obtained via SXES.

## Introduction

Amorphous carbon nitride (a-CN_x_) thin films have attracted considerable attention owing to their unique properties, such as high wear resistivity^1^, low friction coefficient^2,3^, variable optical bandgap^4^, environment-dependent electrical resistance^5^, and biocompatibility^6,7^. Most recently, it was found that a-CN_x_ films exhibit photomechanical response^8,9^. This response manifests as temporal deformation under visible light irradiation. The amount of deformation is maximum in the blue region at around 460 nm in wavelength^10^.

Photomechanical response has been observed in several organic molecules, such as azobenezene^11,12^ and spiropyran^13,14^ and a few inorganic materials^15–17^. Some materials show reversible and irreversible responses to photo-irradiation. Azobenezene shows photomechanical reversible response to photo-irradiation with different photon energy which are UV and visible light, or thermal energy^11,12^. Macroscopic large deformation of films containing ordered the organic molecules has been observed^18^. Piezoceramics, such as Pb_1-x_La_x_(Zr_y_Ti_1-y_)_1-x/4_O_3_ (PLZT), deform corresponding to the switching off and switching on of visible light^15^. This response is a combination effect of photoinduced strain and piezoelectricity. Chalcogenide glasses such as As_2_S_3_ and amorphous Se, show an irreversible response^16,17^. Volume expansion of this material leads to an increase in the defect density induced by photon irradiation.

For carbon-related materials, carbon nanotubes (CNTs) embedded in polymer films^19,20^ have been reported to undergo considerable photoinduced deformation. This underlying mechanism is the photothermal effects of CNTs. Photoinduced deformation of CNTs on a polymer film has also been reported^21^. This deformation can be attributed to the photothermal effects of CNTs and a difference in thermal expansion coefficients between CNTs and a polymer film.

The a-CN_x_ films undergo photoinduced deformation when the temperature of the films increases by less than 1 K^8^. Thus, the photothermal effects in the photomechanical response of a-CN_x_ films would be quite weak. Moreover, the photoinduced deformation has been observed in an a-CN_x_ film with high resistivity and low photoconductivity^22^. From these results, we assume that the origin of photoinduced deformation is the same as that of photoconductivity. That is, the photoinduced deformation of a-CN_x_ films occurs as a result of electron excitation by photo-irradiation.

In this study, we investigate the relationship between photoinduced deformation and surface electrical states by comparing two samples, with different amount of deformation, via scanning electron microscopy with Ar^+^ laser irradiation (SEM-L) and soft X-ray emission spectroscopy (SXES). Sample A was an a-CN_x_, grown at a temperature of 473 K, showed a large amount of photoinduced deformation, while sample B grown at a temperature of 873 K showed a small amount of the deformation. This study is a first report of SEM-L which is a new approach to reveal the mechanisms of photoinduced deformation in all materials with photomechanical response.

## Results

**Figure 1:**
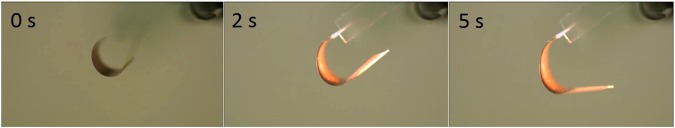
Typical photomechanical response of a-CN_x_ film. The substrate is a 12-μm thick poly(ethylenenaphthalate) film.

The typical photomechanical response of a-CN_x_ films is shown in Fig. 1 (and Supplemental video). A film was deposited on a 12-μm thick poly(ethylenenaphthalate), PEN, film at 373 K. A curvature of the specimen becomes flat by irradiation. For comparing the amount of deformation for samples A and B, a displacement of the free end of the films deposited on Si substrate was measured by using cantilever technique. The magnitudes of photoinduced deformation, δ_y_, in samples A and B were 12.3 and 0.2 μm, respectively.

The photoinduced stress *σ*_p_ was estimated from δy and the following equation^23^:

1 $${\sigma }_{p}=\frac{E{D}^{2}{\delta }_{y}}{3(1-\nu ){L}^{2}d}$$

where *d* and *D* are the thickness of the films and the substrate, respectively. *E* and ν are the Young’s modulus and Poisson ratio of the Si substrate, and their values are 1.3 × 10^11^ Pa and 0.28, respectively. *L* is the distance from the free edge of the sample to the position sensor. The photoinduced stresses in samples A and B were 3.7 and 0.1 MPa, respectively.

**Figure 2:**
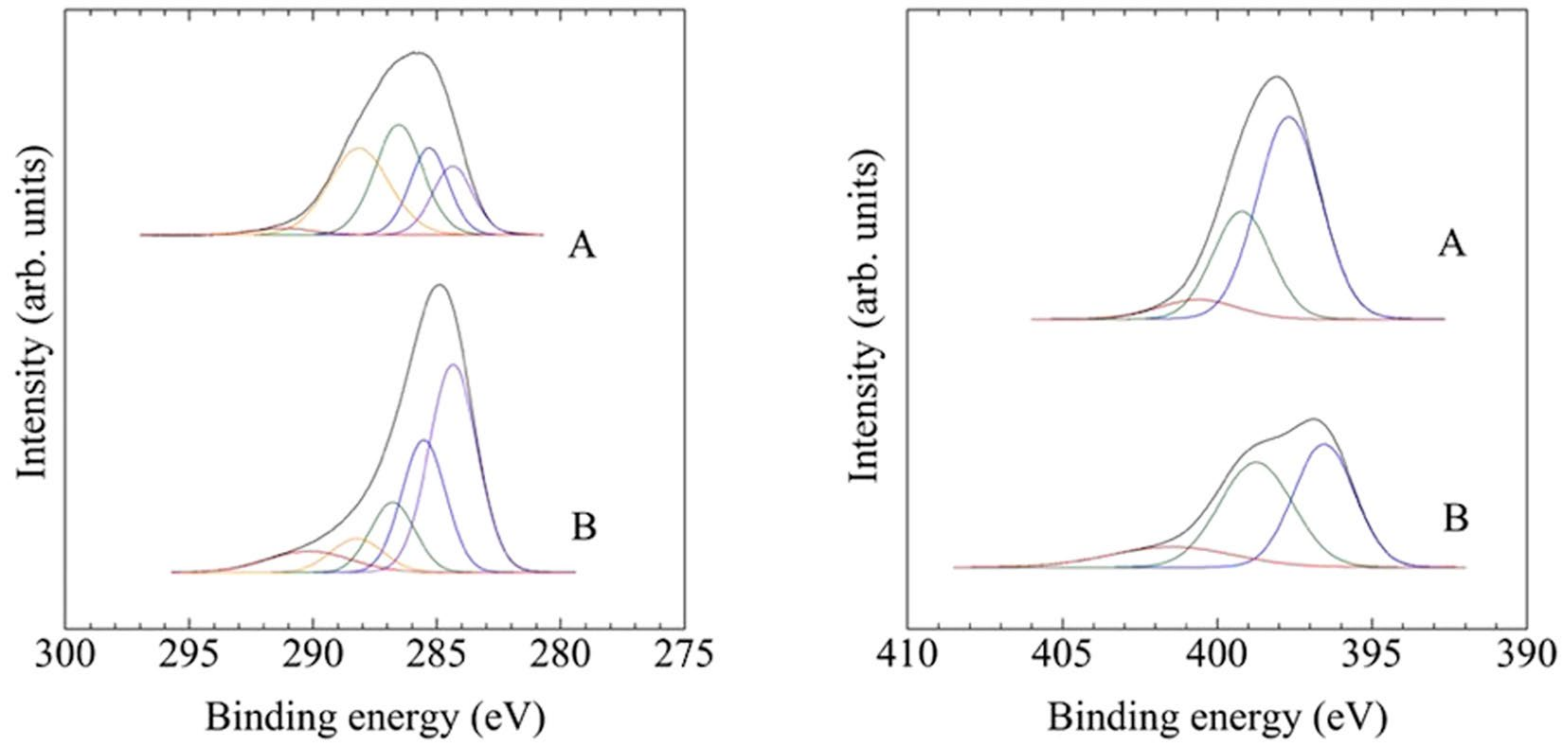
C 1 s and N 1 s XPS spectra of a-CN_x_ films deposited at 473 K (sample A) and 873 K (sample B).

Figure 2 displays C 1 s and N 1 s XPS spectra for both films. C 1 s and N 1 s were separated into their individual chemical bonding components using Gaussian–Lorentzian fitting. The atomic concentration in the films was calculated from the integrated intensity of N 1 s and C 1 s and the photoelectron cross section of core levels. The nitrogen atomic concentration of sample A and sample B were 34.2 at.% (x = 0.52) to 24.2 at.% (x = 0.32), respectively.

**Figure 3:**
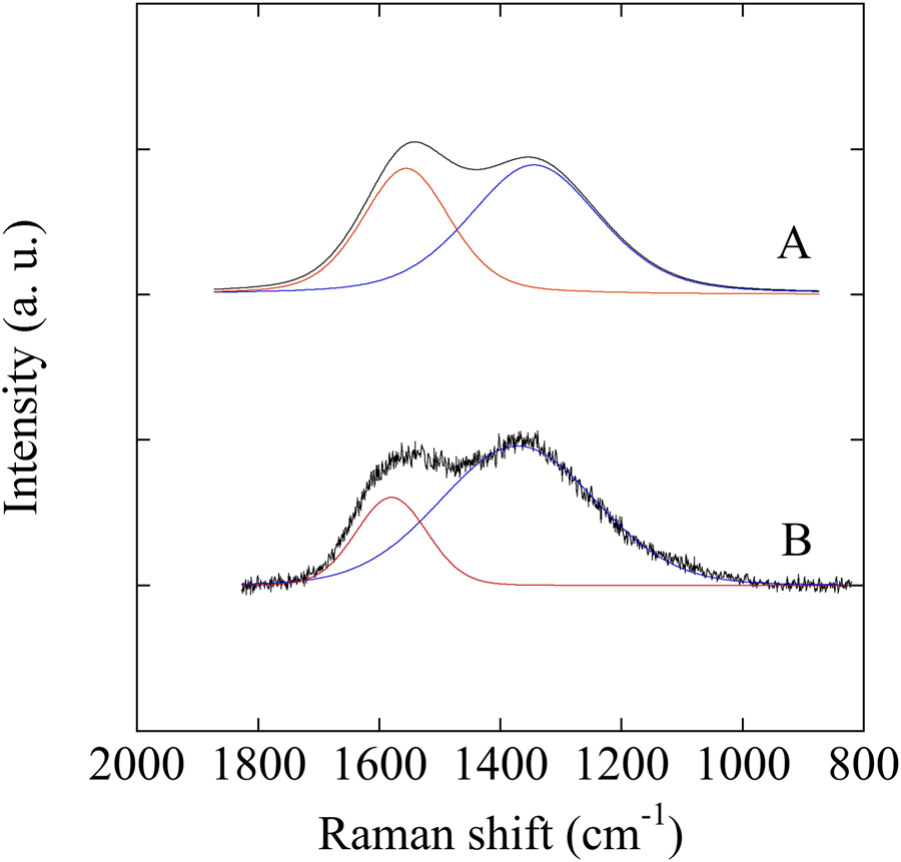
Raman spectra of a-CN_x_ films deposited at 473 K (sample A) and 873 K (sample B).

Figure 3 shows Raman spectra of samples A and B. The Raman spectra contain two peaks called D and G peaks. The G peak at 1500–1600 cm^−1^ arises from the bond stretching motion of pairs of sp^2^C atoms in aromatic rings or olefinic chains. The D band at ~1350 cm^−1^ arises from the breathing modes of sp^2^ atoms in clusters of six-fold aromatic rings^24^. When the graphite cluster size decreases, the intensity of D band *I*_D_ decreases because its internal disorder increases, and the intensity of G peak *I*_G_ remains unchanged because it arises from all sp^2^ stretching modes^25^. Thus, a growing *I*_D_/*I*_G_ ratio is correlated to an increase of graphite. The *I*_D_/*I*_G_ ratios of samples A and B were 2.77 and 3.44, respectively. That means the clusters are larger in sample B compared to sample A.

From XRR analysis, the densities of samples A and B were ~1.6 and ~1.3 g/cm^3^, respectively.

**Figure 4:**
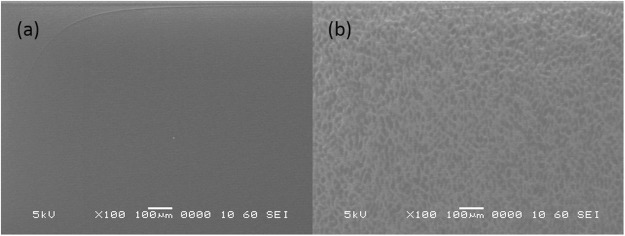
SEM image of a-CN_x_ film with photoinduced deformation; (**a**) without Ar^+^ laser irradiation and (**b**) with irradiation.

To investigate the origin of the photoinduced deformation, SEM-L was performed on samples A and B. The films were deposited on 0.05-mm thick Si substrates. Figure 4 shows the SEM-L secondary electron image of sample A when the laser light is switched on and off. The surface morphology was flat and uniform before light irradiation (Fig. 4a). Owing to irradiation, bright and dark patches that were similar to a speckle pattern appeared on the film surface (Fig. 4b). This inhomogeneous contrast appeared rapidly by irradiation. Subsequently, a few seconds after the light was turned off, the film showed homogeneous contrast as it was before irradiation. This behavior agrees well with the results of time-dependent photoinduced deformation measurement. The average area of the dark spot was ~200 μm^2^. In the case of sample B with small photoinduced deformation, this pattern was hardly visible. The patches were observed on many samples deposited on a 10-μm thick Al foil at different temperatures from 300 to 573 K. It is noteworthy that the speckle-like pattern was observed only in the SEM image of the surface of a-CN_x_ films with photomechanical response.

**Figure 5:**
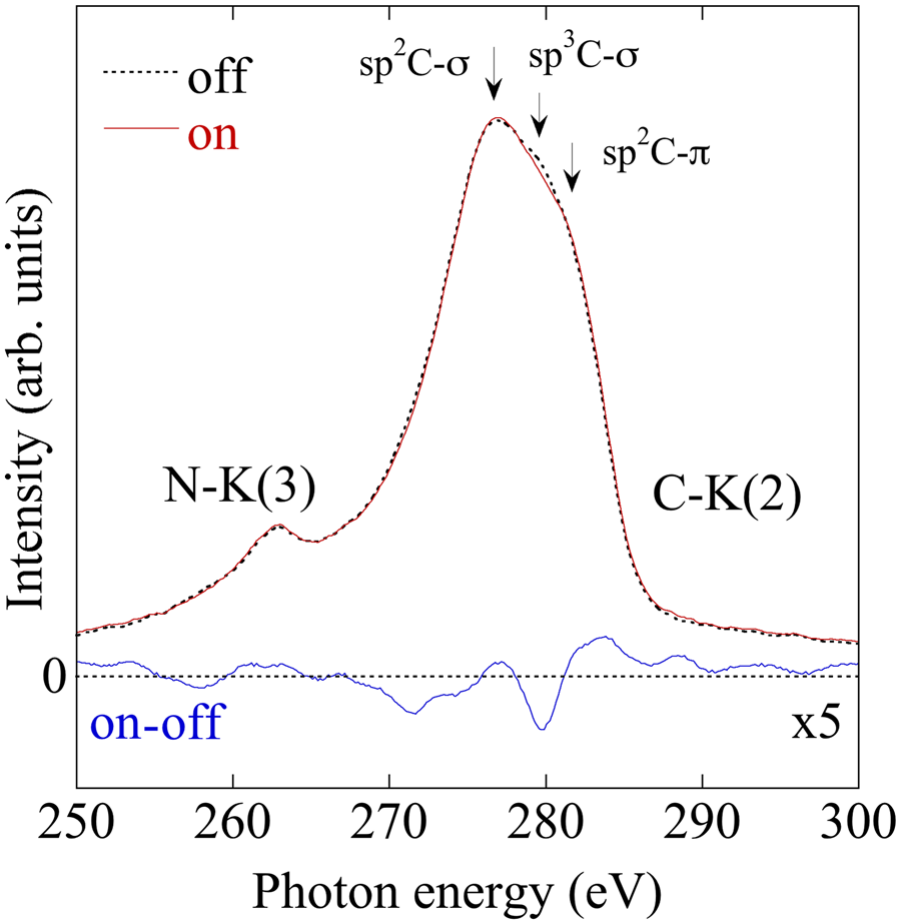
SXES spectra of a-CN_x_ film with photoinduced deformation by turning Ar^+^ laser on and off.

The SXES spectrum, when the laser light is switched on and off, is shown in Fig. 5. This spectrum was obtained using a custom-made SXES system attached to the SEM. An excitation light was the same as SEM-L. The peaks in a second-order carbon K-emission spectrum were assigned to those of graphite and diamond^26^. The presence of sp^2^C-σ bonding is noticed by a structure at 276 eV, which is a graphite characteristic. The presence of sp^3^C-σ bonding is also noticed by a structure at 279 eV, which is a diamond characteristic. Intensity of the peak at 279 eV decreased temporarily under illumination.

## Discussion

a-CN_x_ films have a compressive stress in nature^9^. Thus, a significant bend of a-CN_x_ films that were deposited on flexible PEN substrate can be attributed to the residual stress of compressibility in the a-CN_x_ films. This intrinsic stress evolution in the a-CN_x_ film has been attributed to the structural transformation induced by nitrogen incorporation and by C–C distortions^27,28^.

Even if the structure comprised graphitic clusters, the overall density of the film was significantly below that of graphite (2.27 g/cm^3^)^29^. The densities of samples A and B, as obtained from XRR, were ~1.6 and ~1.3 g/cm^3^, respectively. Low density of a-CN_x_ could be attributed to the high deposition pressure. It can be explained by a low mobility on the surface due to the deposited particles having very low energy.

On XPS spectra, C 1 s core level peaks consisted of five primary components centered at C1 (284.5 eV), C2 (285.2 eV), C3 (286.1 eV), C4 (287.2 eV), and C5 (289.0 eV), respectively. N 1 s core level peaks were decomposed into two main components N1, N2 and N3 centred at 398.3, 400.1, and 402.8 eV, respectively. C1 and C2 peaks are attributed respectively to the chemical state of C atom with CC sp^2^ coordinated bonds in graphite and planner carbon sheet and the chemical state of C atom with CC sp^3^ bonds in diamond and diamond-like amorphous carbon^30,31^. C3 and N2 peaks are attributed to N bonded to trigonal C and the C4 and N1 peaks are attributed to N bonded to tetrahedral C^30^. C5 and N3 are assigned oxygen bonds derived from contamination and surface oxidation after deposition. As shown in Fig. 2a and Table 1, sp^2^CC bond was dominant in sample B. This result reinforces the presence of large graphitic clusters in sample B that will be discussed in Raman spectra. In case of nitrogen bonds, the predominant bonding in a-CN_x_ films changed from N bonded to tetrahedral C to N bonded to trigonal C at an elevated deposition temperature.

A large magnitude of deformation under illumination was obtained for sample A, which contains small graphitic cluster, a large amount of nitrogen, and a high concentration of N bonded to tetrahedral C in comparison with sample B.

For sample A, speckle-like pattern appeared on the film surface during SEM-L observation. In general, random speckle patterns are known to occur owing to the interference of coherent light reflected onto a sample surface^32^. Thus, speckle patterns primarily reflect the surface roughness of a specimen. In terms of surface morphology, the dark and bright parts in SEM images are defined as the concave and convex surfaces of a sample, respectively. However, the surface roughness and morphology of sample A did not change upon irradiation with visible light by using AFM and spectroscopic ellipsometry. In the meanwhile, it is still clear that electron excitation by photon irradiation is relevant to the photoinduced deformation as mentioned above. Therefore, the uniformity of the secondary electron emission of a-CN_x_ was considered because the number of secondary electron generations depends on the element and its density as well as the surface morphology of the samples.

The secondary electron yield of carbon materials decreases with the conversion of sp^3^ hybrids to six-fold aromatic domains, and the underlying reason for this is the strong correlation between the electronic structures close to the Fermi level^33^. If a pattern arises as a result of inhomogeneous secondary electron emission, it indicates that electrons excited by visible light irradiation are localized on the film surface. In fact, the a-CN_x_ films with photomechanical response show little photoconductivity^22^. This experimental fact reinforces the above assumption, which means that the photocarriers generated by visible light are localized in a specific area and behave to change in a structure of the amorphous network.

Our previous study showed an inhomogeneous distribution of the bonding fraction in sample A by means of SXES spectrum mapping measurement using SXES-EPMA system without a visible light source^26^. On a spatial distribution of spectral intensity of sp^2^C, sp^3^C and N in sample A, the intensity ratio of *I*_sp3C_/*I*_sp2C_ for a region of ~30 × 23 μm ranged between 0.89 and 0.94, and large N-content regions had a larger sp^3^C component. The *I*_N_/*I*_sp2C_ of the region ranged between 0.72 and 0.80. These results suggest that a-CN_x_ films have specific area of high intensity of sp^3^C and N in one sample. A specific area of a high intensity of *I*_sp3C_/*I*_sp2C_ exists in sample A and, the dimensions of this specific area is just about coincident with the dimensions of the pattern in SEM-L.

From SXES spectrum, as shown in Fig. 5, an intensity of sp^3^C-σ bonding decreased temporarily under illumination. This result is one of the evidences that photoinduced deformation is caused by temporal and local change of chemical bonds. The temporarily decrease in sp^3^C bonds by photo irradiation suggests a change of the bond angle and bond length variations consistent with energy minimization. Consequently, the films are deformed under illumination.

In summary, we presented a correlation between surface electrical states and deformation of a-CN_x_ films due to visible light irradiation. Two samples with different amounts of photoinduced deformation were prepared by reactive rf magnetron sputtering. A large magnitude of deformation under illumination was obtained in the film with a large nitrogen concentration, high film density and small graphitic cluster. On this film, bright and dark patches, similar to a speckle pattern, appeared on the film surface upon irradiation with an Ar^+^ laser, as observed by SEM. A size of the patches is well correlated with an inhomogeneous distribution of sp^2^C, sp^3^C and N. This anomalous behavior has not been reported to date.

### Methods Deposition of a-CN_x_ films

a-CN_x_ films were deposited via reactive radio frequency (rf) magnetron sputtering. The target was a graphite disc (Kojundo Chemical Laboratory, Lot. #4087341; purity = 99.995%, diameter ~ 5 cm), and only N_2_ (purity = 99.9995%) was used as the sputtering gas. Total N_2_ gas pressure was 16 Pa. Frequency and rf power and frequency were 13.56 MHz and 85 W, respectively. The target-substrate distance was approximately 4 cm. A Si(100) substrate with 0.5 mm thickness was used for the characterizations except photoinduced deformation. To investigate the relationship between photoinduced deformation and surface electrical states, two samples with different amount of deformation were deposited on Si substrate (length, 20 mm; width, 2 mm; and thickness, 0.05 mm). Samples A with a large amount of the deformation was deposited at 473 K and sample B with a small amount of the deformation was deposited at 873 K. a-CN_x_ film was also deposited on a 12-μm thick poly(ethylenenaphthalate) film and a 10-μm thick Al foil. In the proposed study, the total thickness of all a-CN_x_ films was ~1 μm.

### Measurements and characteristics

To evaluate the nitrogen atomic concentration and chemical bonding states of the films, X-ray photoelectron spectroscopy (XPS; KRATOS ULTRA2, Shimadzu) was performed using Al monochromatic X-rays (1486.6 eV). The pass energy and step size was 40 eV and 0.05 eV, respectively.

Raman spectroscopy (NRS-5100 laser Raman Spectrometer, JASCO) was employed to obtain the hybridization states of the films. An Ar^+^ laser (λ = 532.1 nm) with a power of 0.5 mW was used as the source of the excitation light.

The film densities were calculated using the critical angle θ_c_ of the total X-ray reflection determined via X-ray reflectivity (XRR; SmartLab, Rigaku) performed with Cu-Kα (λ = 0.15406 nm) monochromatic radiation, which was the scanning speed was set to 1°/min.

The a-CN_x_ film thickness was measured using a field emission scanning electron microscope (FE-SEM; S-4500, Hitachi). The acceleration voltage and work distance was set to be 15 kV and 15 mm, respectively. The films were deposited on Si(100) substrate with a thickness 0.5 mm and then its fracture cross section was observed using FE-SEM.

**Figure 6:**
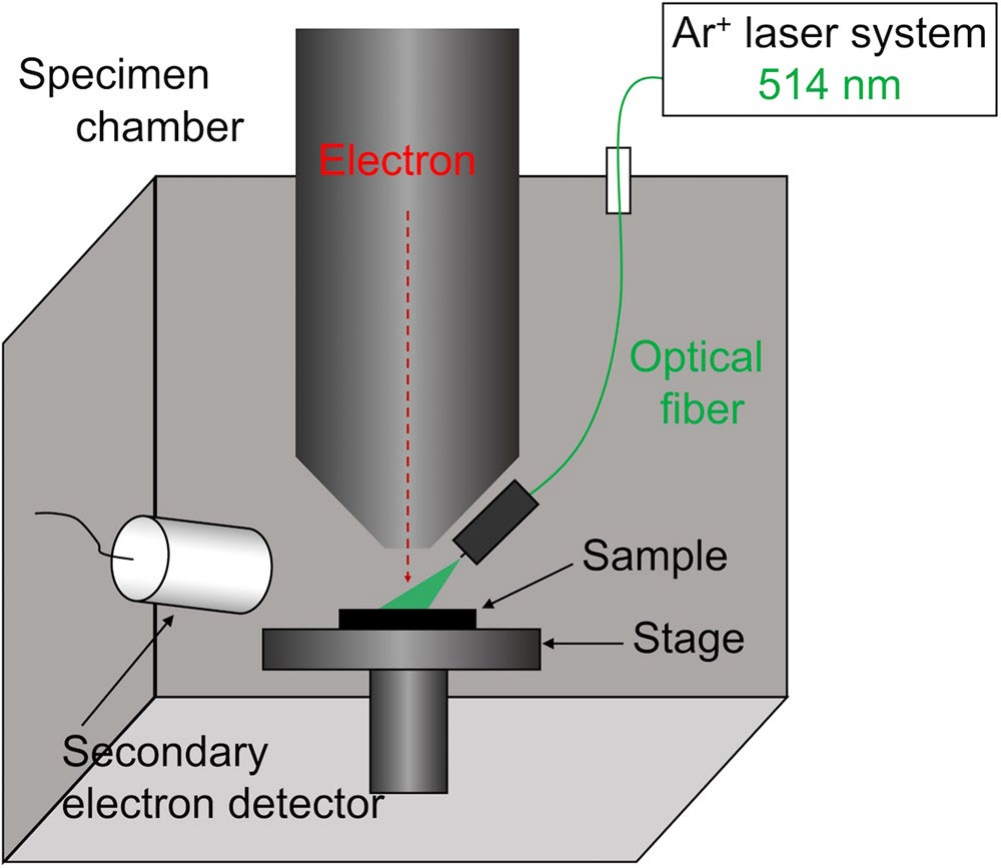
A schematic image of SEM-L system.

The SEM-L apparatus used in this study was based on JEOL JSM6480LV. The laser light was irradiated on the sample surface through optical fiber, which was attached to the chassis of electron probe. The schematic image of SEM-L was shown in Fig. 6. The acceleration voltage was set to be 5 kV. The film is deformed by irradiation of visible light from 350 to 750 nm^10^. Hence Ar^+^ laser (λ = 514 nm) was used for exciting of the films.

Two SXES systems were used for obtaining the second-order spectrum of C K-emission, C-K(2), and third-order N K-emission spectrum, N-K(3). One was a commercial SXES system (SS-98000, JEOL) attached to an electron probe microanalyser (EPMA; JXA-9800, JEOL), SXES-EPMA. The probe current and acceleration voltage of this device were set to 40 nA and 5 kV, respectively. The other was a custom-made SXES system attached to a SEM, SXES-SEM^34^. The probe current and acceleration voltage of this device were set to 120 nA and 5 kV. The spectra represent as C-K(1) emission energy, which is the real X-ray energy emitted from the specimen of ~280 eV. A peak intensity around 265 eV corresponds to third-order nitrogen K-emission, N-K(3), whose true X-ray energy is ~395 eV^26^.

The amount of photoinduced deformation was measured using a rectangular specimen and the optical-lever technique. The substrate was Si with 20 mm in length, 2 mm in width and 0.05 mm in thickness. The measurement system is described in ref.^8^. One end of the specimen was held on tight to a specimen holder like a cantilever, and the other free end of the specimen was measured using a He–Ne laser. A white light from Xe lamp through IR cut filter was used as the excitation light source.

Atomic force microscopy (AFM; SPM-9700, Shimadzu) and spectroscopic ellipsometry (alpha-SE, J.A. Woollam) were also carried out under visible light illumination to clarify the cause of photoinduced deformation. For both methods, the excitation light was irradiated to the surface of the films through optical fiber from a Xe lamp.

**Table 1:** Bonding components of a-CN_x_ films.

sample	C 1 s				N 1 s	
	C1 (284.5 eV)	C2 (285.2 eV)	C3 (286.1 eV)	C4 (287.2 eV)	N1 (398.3 eV)	N2 (400.1 eV)
A	16.3%	19.7%	31.0%	30.8%	54.6%	42.5%
B	30.1%	36.5%	13.8%	14.5%	48.7%	48.4%

## Electronic supplementary material

Supplemental video
